# Identification of a mesenchymal-related signature associated with clinical prognosis in glioma

**DOI:** 10.18632/aging.202886

**Published:** 2021-04-19

**Authors:** Zhengwei Zhang, Jie Chen, Xiuhao Huo, Gang Zong, Kebing Huang, Meng Cheng, Libo Sun, Xiaoyu Yue, Erbao Bian, Bing Zhao

**Affiliations:** 1 Department of Neurosurgery, The Second Affiliated Hospital of Anhui Medical University, Hefei 230601, China; 2 Cerebral Vascular Disease Research Center, Anhui Medical University, Hefei 230601, China

**Keywords:** glioma, mesenchymal, gene signature, prognosis, proliferation

## Abstract

Malignant glioma with a mesenchymal (MES) signature is characterized by shorter survival time due to aggressive dissemination and resistance to chemoradiotherapy. Here, this study used the TCGA database as the training set and the CGGA database as the testing set. Consensus clustering was performed on the two data sets, and it was found that two groups had distinguished prognostic and molecular features. Cox analysis and Lasso regression analysis were used to construct MES signature-based risk score model of glioma. Our results show that MES signature-based risk score model can be used to assess the prognosis of glioma. Three methods (ROC curve analyses, univariate Cox regression analysis, multivariate Cox regression analysis) were used to investigate the prognostic role of texture parameters. The result showed that the MES-related gene signature was proved to be an independent prognostic factor for glioma. Furthermore, functional analysis of the gene related to the risk signature showed that the genes sets were closely related to the malignant process of tumors. Finally, FCGR2A and EHD2 were selected for functional verification. Silencing these two genes inhibited the proliferation, migration and invasion of gliomas and reduced the expression of mesenchymal marker genes. Collectively, MES-related risk signature seems to provide a novel target for predicting the prognosis and treatment of glioma.

## INTRODUCTION

Glioma mainly occurs in the brain and glial tissue, and is the most common primary malignant brain tumor in adults [1, 2]. Gliomas are astrocytes, oligodendrocytes, or a mixture of these two cell types. Gliomas are classified into four categories (grades I~IV) based on malignancy and overall survival (OS) by the International Classification of Diseases–Oncology, version 3 (ICD-O-3) and the World Health Organization (WHO). Among them, grade IV glioma (glioblastoma) has a very poor prognosis, with a median survival between 14.5 and 16.6 months [3, 4]. Even after surgery, radiotherapy and chemotherapy, the survival time of patients with glioma is only extended by a few months [5]. Therefore, there is an urgent need for accurate prognostic prediction and new therapeutic targets for glioma treatment.

Glioblastoma is the most common primary malignancy in the central nervous system and is fast-growing (grade 4 glioma) [6, 7]. The new classification of GBM (glioblastoma multiforme) tumor subtypes is based on The Cancer Genome Atlas (TCGA) on related genetic mutations, changes in the associated recurrent gene copy number and comprehensive genome sequence analysis. GBM is divided into proneural, neural, classical and mesenchymal subtypes according to different biological, imaging and clinical characteristics [8, 9]. High-grade glioma (HGG) can also be divided into three subtypes: proneural (PN), mesenchymal (MES) and proliferative (Prolif) because of their different molecular characteristics, including CHI3L1 / YKL40, SERPINE1 and PDPN. The MES subtype is a more malignant form with a higher tendency for relapse, metastasis, and increased vascularity [10–12]. MES related to a consistently poor prognosis with a median survival time of 1.2 years [11]. GBM relapse has been verified to be closely associated with mesenchymal, stem-like phenotypes that are resistant to treatment [13, 14]. Mesenchymal GBMs showed the highest percentage of microglia, macrophage, and lymphocyte infiltration, which was connected with a worse prognosis [15].

In recent years, central nervous system tumors have been reclassified, which has produced a paradigm shift in personalized therapeutics and prognostic factor-guided treatment decisions [6]. Recently, it has been proposed that the prognosis of glioma is closely related to immune-related lncRNAs, N6- methyladenosine-related lncRNAs and energy metabolism-related genes [16–18]. Therefore, we speculate that the detection of MES-related genes is of great significance for evaluating prognosis. In addition, these findings may contribute to the discovery of prognostic biomarkers for glioma and the development of more accurate treatment processes.

This study used TCGA and Chinese Glioma Genome Atlas (CGGA) RNA sequencing data to study the clinical value of MES-related genes from the TCGA and Ivy Glioblastoma Atlas Project (Ivy GAP) databases. According to the expression levels of MES-related genes, the patients were divided into two groups by consensus clustering analysis, and there were significant differences in prognosis and molecular characteristics. Then, Lasso regression was used to calculate the regression coefficient, and the risk score was calculated based on the regression coefficient and the gene expression level. The patients were graded into high-risk and low-risk groups of the median risk score, in which the low-risk group had a better prognosis than the high-risk group. The MES-related signature is closely related to the prognosis of patients and could act as an independent pathological predictive factor. Furthermore, functional analysis showed that gliomas with a higher risk score for MES-related genes were associated with many aspects of glioma progression, including epithelial-mesenchymal transition, angiogenesis, hypoxia and inflammatory response. Subsequent cell function test results showed that inhibition of FCGR2A or EHD2 significantly represses proliferation, migration, and invasion of glioma cells, and reduces the expression of mesenchymal marker genes. Therefore, these results suggest that MES-related genes will be better for predicting the prognosis of glioma and provide novel targets for glioma treatment.

## RESULTS

### Fifteen MES-related genes from the TCGA and Ivy GAP databases

**Figure 1:**
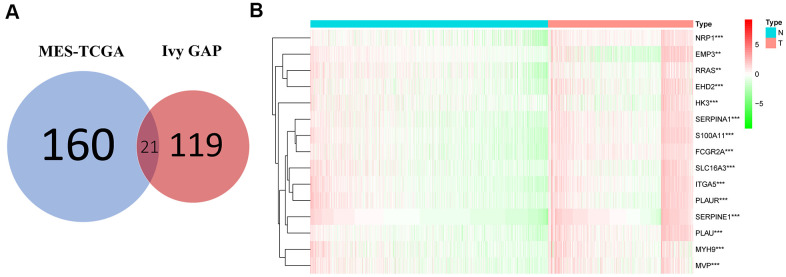
**Acquisition and verification of MES-related genes.** (**A**) Venn diagram indicating that 21 MES-related genes were selected from the Ivy GAP and TCGA databases. (**B**) The heat map shows that 15 of the MES-related genes were significantly different between the normal and tumor groups. **P <. 01; ***P < 0.001.

The MES-related genes were obtained from the TCGA and Ivy GAP databases. As shown in Figure 1A, the Venn diagram showed a total of 21 intersecting genes, including EFEMP2, CHI3L1, TIMP1, EMP3, NRP1, EHD2, HK3, RRAS, FES, PTRF, MYH9, MVP, SERPINA1, S100A11, THBD, FCGR2A, SLC16A3, ITGA5, and PLAU. Then, we used differential gene analysis to identify differentially expressed genes between the normal and tumor groups, 15 of which had significant differences between the two groups, including NRP1, EMP3, FCGR2A, PLAUR, SERPINA1, SERPINE1, MYH9, S100A11, MVP, RRAS, ITGA5, SLC16A3, EHD2, PLAU, and HK3, and all had a positive correlation (Figure 1B and Table 1).

**Table 1:** Full names of the 15 related genes and their p-values.

**Gene symbol**	**Gene name**	**p value**
SERPINE1	Serpin Family E Member 1	9.48E-98
S100A11	S100 Calcium Binding Protein A11	8.36E-82
ITGA5	Integrin Subunit Alpha 5	3.54E-69
HK3	Hexokinase 3	3.55E-04
RRAS	RAS Related	9.32E-03
FCGR2A	Fc fragment of IgG receptor IIa	2.84E-108
PLAU	Plasminogen Activator, Urokinase	2.72E-114
SERPINA1	Serpin family A member 1	1.53E-74
MYH9	Myosin Heavy Chain 9	1.44E-47
NRP1	Neuropilin 1	2.71E-71
PLAUR	Plasminogen Activator, Urokinase Receptor	1.73E-23
EHD2	EH Domain Containing 2	1.87E-30
EMP3	Epithelial Membrane Protein 3	2.35E-03
MVP	Major Vault Protein	1.00E-39
SLC16A3	Solute Carrier Family 16 Member 3	2.29E-32

### Stratification of gliomas based on the fifteen MES-related genes

**Figure 2:**
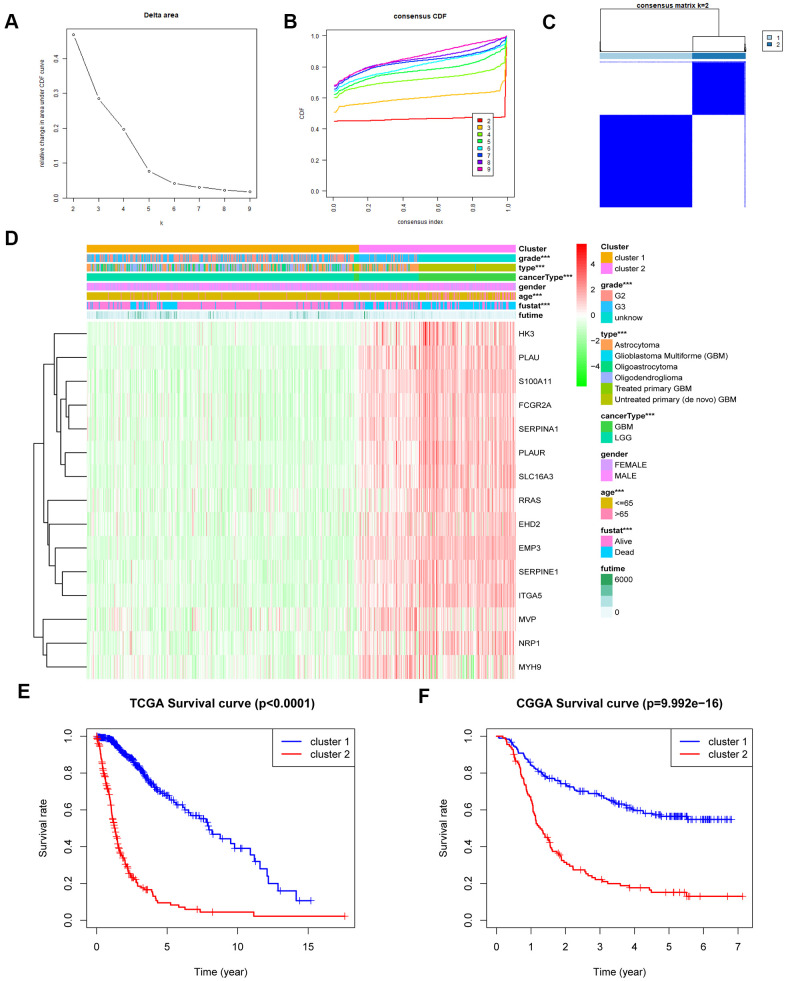
**MES-related gene sets could classify the clinical and molecular features of gliomas.** (**A**) Relative change in the area under the CDF curve for k = 2 to k = 9. (**B**) Consensus clustering CDF for k = 2 to k = 9. (**C**) Consensus clustering matrix of 650 samples from the TCGA dataset for k = 2. (**D**) Heat map of MES-related genes between cluster 1 and cluster 2 of the TCGA cohort. (**E**) Survival analysis of patients in cluster 1 and cluster 2 based on TCGA clinical data. (**F**) Survival analysis of patients in cluster 1 and cluster 2 based on CGGA clinical data. CDF, cumulative distribution function; ***P <0.001.

Consensus clustering of 650 samples identified two clusters. In the TCGA data set, the cluster stability increased between k = 2 and k = 9. Next, we observed significant differences in the clinical and molecular characteristics of the two clusters identified by consensus clustering (Figure 2A–2C and Supplementary Figure 2A). In the training cohort, cluster 1 was closely related to the age at diagnosis and grade of glioma, as well as the type of glioma (p<0.001) (Figure 2D). Similarly, these results were verified in the CGGA database (Supplementary Figure 2B–2F). In addition, we observed that the overall survival (OS) of cluster 2 was significantly shorter than cluster 1 (Figure 2E). At the same time, the CGGA validation set clearly showed the same results in the two different prognostic subgroups (Figure 2F). These results indicate that the MES-related gene set is related to the survival time of patients with glioma, and cluster 2 is much shorter than cluster 1.

### Establishment of the MES-related gene risk signature

**Figure 3:**
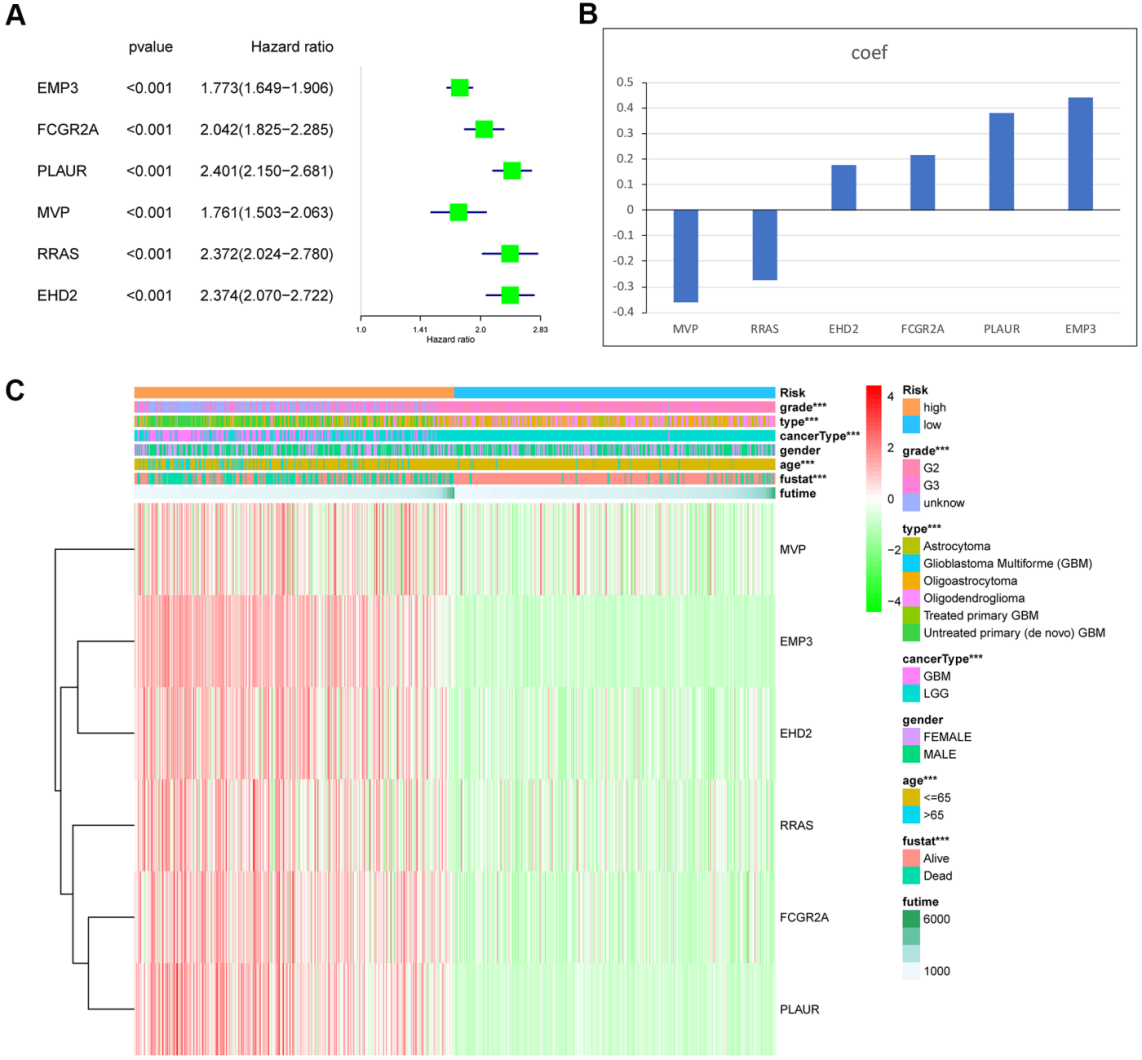
**Identification of the 6-gene risk signature by Lasso regression analysis in the TCGA dataset.** (**A**) The hazard ratio and P value of the 6 MES-related genes. (**B**) Coefficient values for each of the 6 selected genes. (**C**) Heat map showing the association of risk scores and clinicopathological features. ***P < 0.001.

The predictive value of the risk score model was evaluated using the TCGA data set as the training set. To establish the MES-related gene signature, first, univariate Cox regression analysis of all data from the training and testing cohorts was used to select six genes from 15 MES-related genes. We chose six genes as risk coefficients in the model because these six genes had higher hazard ratios (Figure 3A). Then, through the Lasso regression algorithm, six genes were designated as active covariates to gauge the prognostic value and obtain the correlation coefficients of each gene (Figure 3B). Next, the risk score of the patients was calculated by the correlation coefficients and gene expression. The training set was segmented into high-risk and low-risk groups based on their median risk score to verify the performance of the risk score as a classifier for evaluating characteristic genes. We found clinically significant differences and different molecular characteristics between the high- and low-risk groups (Table 2). The high-risk group had an older age, a higher glioma grade, and a more malignant glioma type than the low-risk group (Figure 3C).

**Table 2:** Correlation between 6-gene-based risk scores and clinicopathological factors of glioma patients in the two cohorts.

**Training set RNA-seq cohort (n =665)**			
**Features**	**Low-risk score**	**High- risk score**	**P- value**
	**n=333**	**n=332**	
Age			
≤ 65	320	258	**<0.0001**
> 65	13	74	
Gender			
Male	189	194	ns
Female	144	138	
Grade			
II	186	59	**<0.0001**
III	145	115	
IV	2	158	
Vital status			
Alive	294	152	**<0.0001**
Dead	39	180	
**Testing set RNA-seq cohort(n=280)**			
**Features**	**Low-risk score**	**High- risk score**	**P- value**
	**n=165**	**n=115**	
Age			
≤ 65	163	112	ns
> 65	1	2	
Gender			
Male	96	71	ns
Female	69	44	
Grade			
II	98	13	**<0.0001**
III	30	22	
IV	37	77	
Vital status			
Alive	90	21	**<0.0001**
Dead	75	94	

The training set RNA-seq cohort comes from the TCGA database and testing set RNA-seq cohort comes from the CGGA database. Ns: no significance; Bold type indicates a statistically significant difference (P value < 0.05).

To verify whether the MES-related risk characteristics are equally applicable in another sample, we developed a risk score for each patient in the CGGA database according to the risk score of the training set. Consistent with the above results, compared with low-risk groups, high-risk groups tend to have more malignant clinical features. In general, we still found important differences between the two clusters in the independent verification group (Supplementary Figure 3A–3C).

### The 6-gene signature shows strong prognostic power

**Figure 4:**
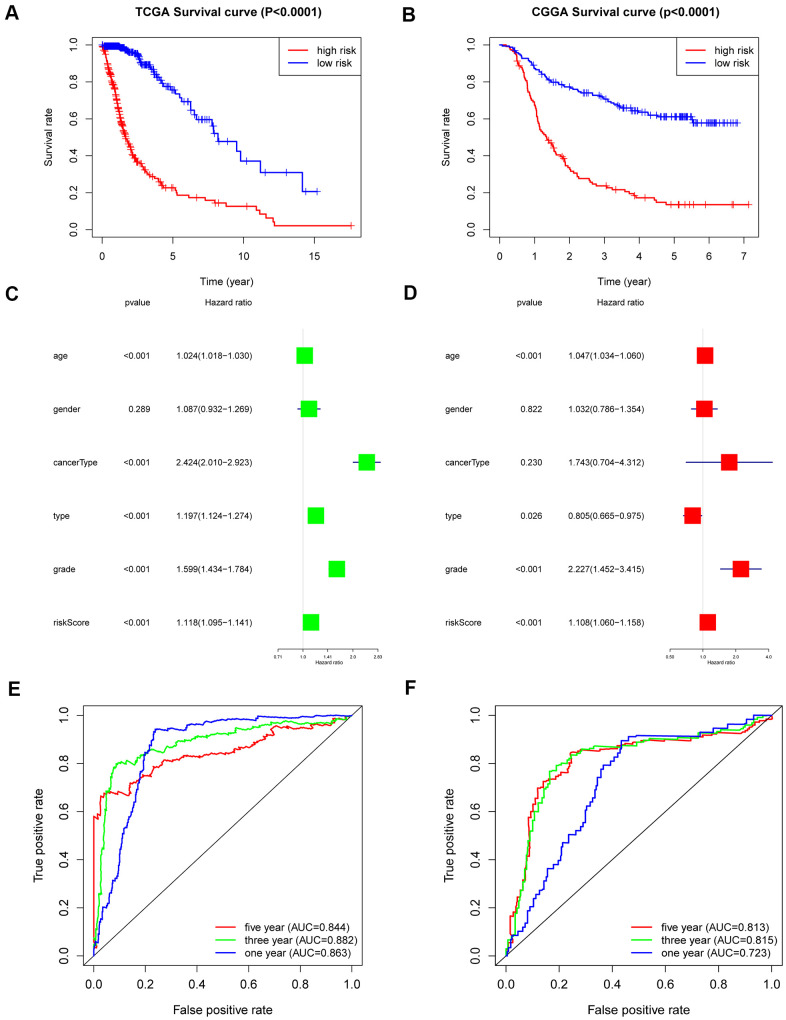
**Outcome prediction of the 6-gene signature in stratified patients of the TCGA cohort and CGGA cohort.** (**A**) Kaplan-Meier overall survival analysis between the high- and low-risk groups in the TCGA cohort. (**B**) Kaplan-Meier overall survival analysis between the high- and low-risk groups in the CGGA cohort. (**C**, **D**) Univariate (**C**) and multivariate Cox regression (**D**) analyses of clinical features and the 6-gene-based risk score for OS in the TCGA dataset. (**E**) ROC curves indicating the sensitivity and specificity of predicting 1-, 3- and 5-y survival with the MES-related signature in the TCGA dataset. (**F**) ROC curves indicating the sensitivity and specificity of predicting 1-, 3- and 5-y survival with the MES-related signature in the CGGA dataset.

The overall survival rate of patients in the low-risk group was significantly longer than that of patients in the high-risk group by Kaplan-Meier analysis (Figure 4A). In addition, patients in the low-risk group had a significantly positive progression-free survival time compared to the high-risk group (Supplementary Figure 4A). To verify whether the risk coefficient is an independent prognostic factor for the prognosis of glioma, we carried out univariate and multivariate Cox regression analyses. The risk score was not associated with age, sex, grade, or subtype, but it was significantly related to the patient’s OS and was an independent prognostic factor (Figure 4C, 4D). Additionally, the ROC curve was used to further evaluate the specificity and sensitivity of the risk score as a predictor by calculating the AUCs (areas under the curve) of the risk score for 1-, 3- and 5-year OS. The AUC values of the risk score were 0.844, 0.882 and 0.863, which showed its powerful ability to predict prognosis (Figure 4E).

In CGGA, the same results were obtained. Overall survival analysis showed that low-risk patients also had a better prognosis (Figure 4B). We performed univariate and multivariate Cox regression analyses of the CGGA data set, which revealed that the risk score was as an independent prognostic factor (Supplementary Figure 4B, 4C). The ROC curve analysis also showed good specificity and sensitivity for one-, three-, and five-year survival predictions, and the one-, three-, and five-year AUC values were 0.813, 0.815 and 0.723, respectively (Figure 4F).

### Functional analysis of the 6-MES related genes

**Figure 5:**
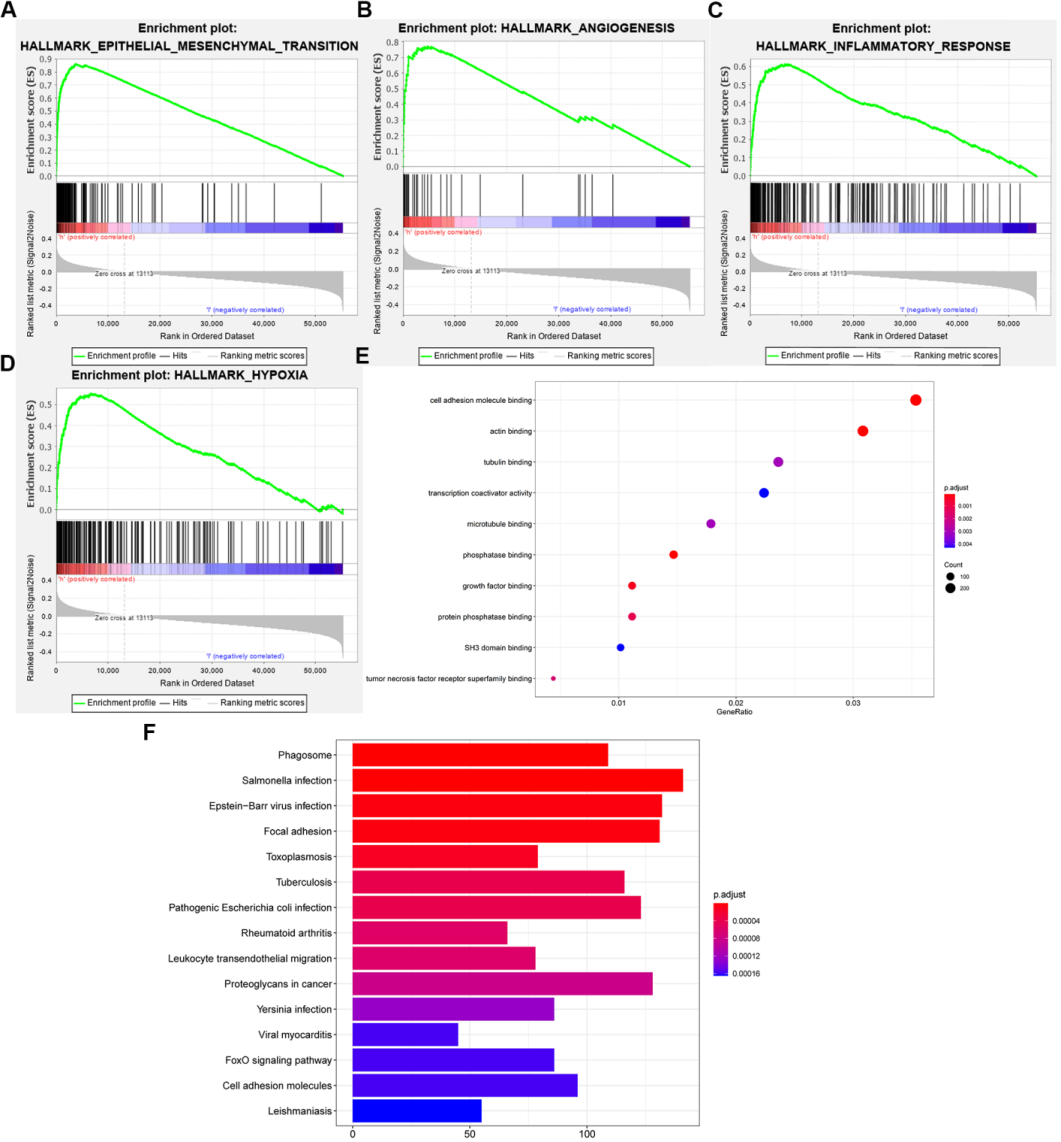
**Functional analysis of the 6-gene signature.** (**A**–**D**) GSEA revealed that the two cohorts were enriched for hallmarks of malignant tumors. (**E**) GO annotations based on the top 4400 genes positively associated with the 6-gene signature. (**F**) KEGG pathways associated with the risk score.

To explore the functional characteristics of potential changes associated with the 6-gene signatures, gene set enrichment analysis (GSEA) was performed between the high- and low-risk score groups. We discovered that the high-risk group was closely related to epithelial-mesenchymal transition, angiogenesis, inflammation, and hypoxia compared to the low-risk group (Figure 5A–5D). Additionally, we performed GO analysis to explore the correlation of the difference function between the high- and low-risk groups. Similarly, we discovered that the positively related genes were mainly enriched in cell adhesion molecule binding, tumor necrosis factor receptor superfamily binding, and tubulin binding (Figure 5E), which are closely associated with the epithelial-mesenchymal transition process. Next, KEGG analysis verified that the high-risk group was closely related to focal adhesion and cell adhesion molecules (Figure 5F). We found that these genes may be involved in these processes. Leading to a worse prognosis for patients with glioma.

### Inhibiting FCGR2A or EHD2 expression could significantly suppress glioma proliferation, migration, and invasion

**Figure 6:**
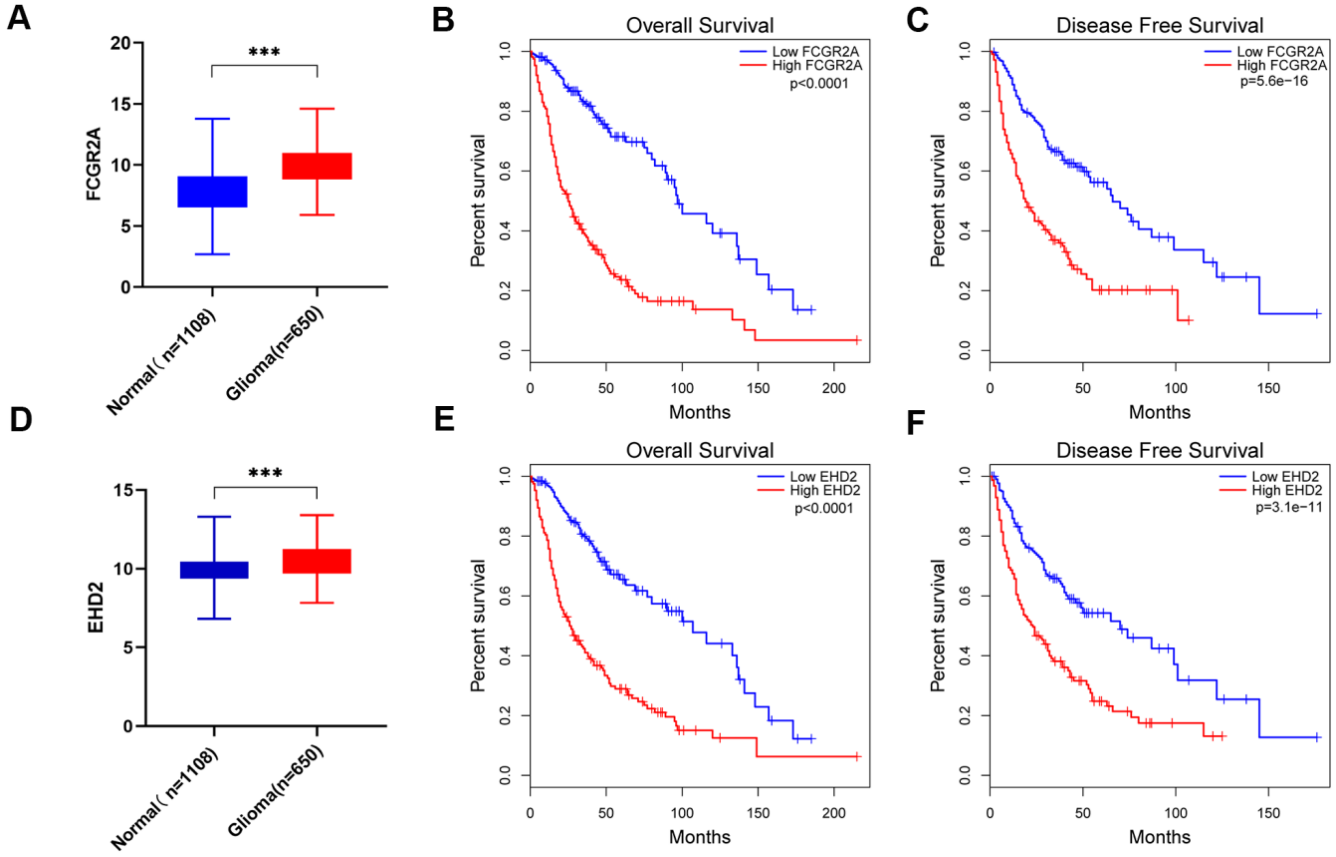
**FCGR2A and EHD2 were selected from the six MES-related genes.** (**A**) Differences in FCGR2A expression between the normal group and the glioma group from the TCGA and GTEx data sets. (**B**, **C**) Overall survival analysis (**B**) and disease-free survival analysis (**C**) of the relationship between FCGR2A expression level and survival time from the TCGA database. (**D**) Differences in EHD2 expression between the normal group and the glioma group from the TCGA and GTEX data sets. (**E**, **F**) Overall survival analysis (**E**) and disease-free survival analysis (**F**) of the relationship between EHD2 expression level and survival time from TCGA database. ***P<0.001.

**Figure 7:**
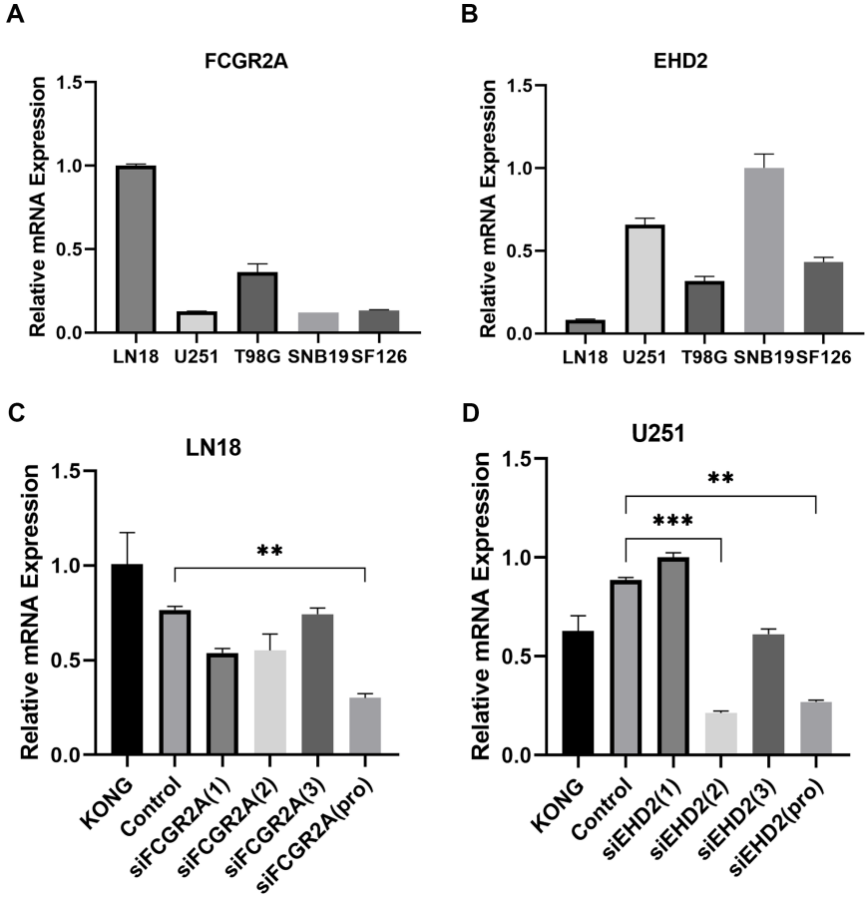
**Selection of cell lines and verification of the silencing effect.** (**A**) Relative expression of FCGR2A in five cell lines. (**B**) Relative expression of EHD2 in five cell lines. (**C**) The LN18 cell line was transfected with three siRNA fragments separately and in combination (pro), and the relative silencing level of FCGR2A. (**D**) The U251 cell line was transfected with three siRNA fragments separately and in combination (pro), the relative silencing level of EHD2. KONG stands for untreated cell; Control stands for Negative control group. **P<0.01; ***P<0.001.

**Figure 8:**
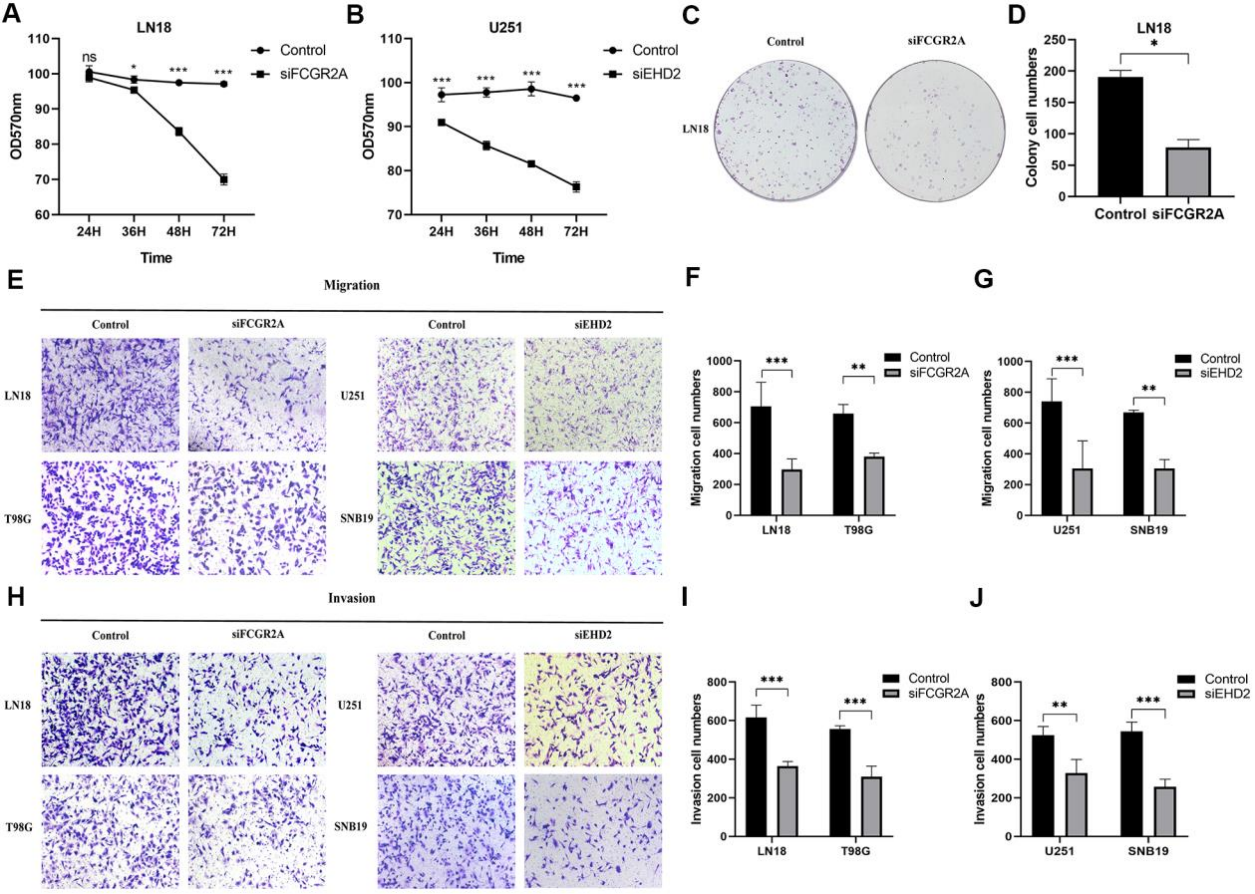
**The effect of glioma cell clone, proliferation, migration and invasion ability after silencing FCGR2A or EHD2.** (**A**, **B**) Cell proliferation was measured by the MTT assay for 24 hours up to 72 hours. (**C**, **D**) Representative imaging (**C**) or counting (**D**) of the colonies formed by LN18 cells after silencing with FCGR2A for 7 days. (**E**–**G**) Representative imaging (**E**) or counting (**F**, **G**) of migration assays after silencing FCGR2A and EHD2 in glioma cells. (**H**–**J**) Representative imaging (**H**) or counting (**I**, **J**) of invasion assays after silencing FCGR2A and EHD2 in glioma cells. *P<0.05; **P<0.01; ***P<0.001.

**Figure 9:**
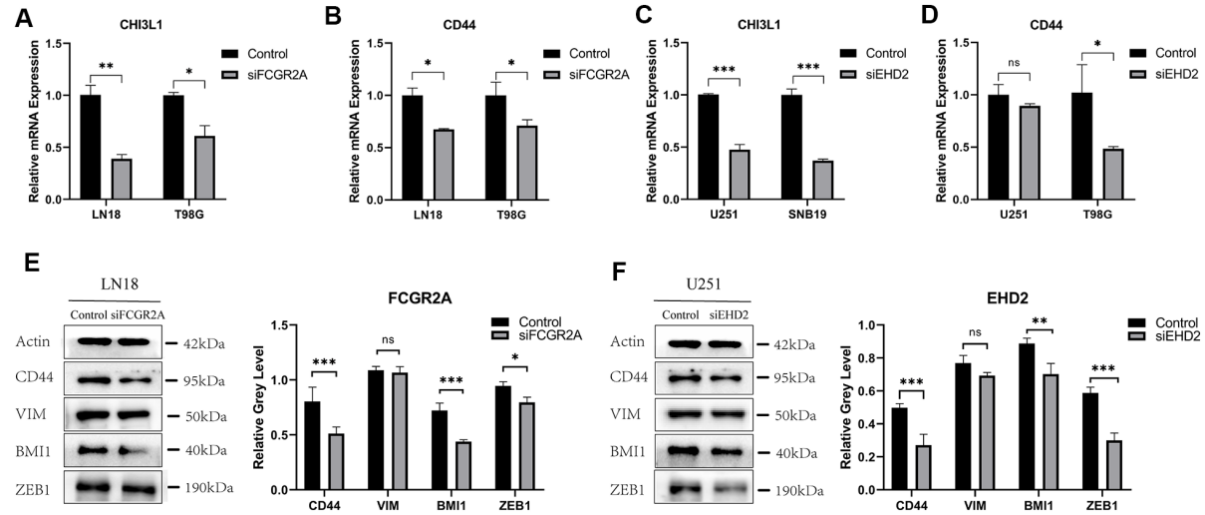
**The relative expression of GBM mesenchymal markers and MES protein markers after FCGR2A or EHD2 silencing.** (**A**, **B**) The relative mRNA expression of CHI3L1 and CD44 after silencing FCGR2A. (**C**, **D**) The relative mRNA expression of CHI3L1 and CD44 after silencing EHD2. (**E**) The western blot analysis of CD44, BMI1, VIM and ZEB1 protein markers after silencing FCGR2A. (**F**) The western blot analysis of CD44, BMI1, VIM and ZEB1 protein markers after silencing EHD2. Ns: no significance; *P<0.05; **P<0.01; ***P<0.001.

Using the database to analyze the gene differences between the normal group(n=1108) and glioma group(n=650), it was also found that these six genes are different except for EMP3. Next, TCGA database was utilized to analyze the overall survival and disease-free survival rate of the six genes between the high-expression group and the low-expression group. We found that glioma patients with high expression levels of these genes had a worse prognosis than those with low expression levels (Figure 6 and Supplementary Figure 5). Subsequently, we analyzed these six genes and found that among them, FCGR2A and EHD2 have rarely been studied in glioma, and there are no functional tests. Hence, we chose these two genes for functional analysis in glioma. Using five existing glioma cell lines in the research group to carry out qRT-PCR, it was found that FCGR2A was expressed at a relatively high level in LN18 and T98G cells, whereas EHD2 was expressed at a high level in U251 and SNB19 cells (Figure 7A, 7B). To verify the effects of these two genes on the proliferation, migration and invasion of glioma cells, the genes were silenced with siRNAs. Three fragments of siFCGR2A (siFCGR2A(pro)) and siEHD2-2 were selected to silence genes in LN18 and U251 cells (Figure 7C, 7D) and were verified in T98G and SNB19 cells (Supplementary Figure 6A, 6B). Functional tests were performed on the cell lines corresponding to these two genes, including the MTT assay, colony formation assay, Transwell assay and Matrigel invasion assay. The MTT assay showed that cell proliferation viability was significantly inhibited at 24 h, 36 h, 48 h and 72 h after transfection (Figure 8A, 8B and Supplementary Figure 7A, 7B). After the colony formation assay was carried out for approximately ten days, it was found that in glioma cells, the cloning ability of cells strikingly decreased after silencing, but it was difficult for U251 cell lines to form colonies (Figure 8C, 8D and Supplementary Figure 7C–7F). The Transwell and Matrigel invasion assays showed that glioma cells' ability to migrate and invade was significantly reduced after siRNA silencing of the target gene (Figure 8E–8J). These results revealed that silencing FCGR2A or EHD2 reduced glioma cell proliferation, migration and invasion. Through mechanistic studies, it was found that silencing the FCGR2A or EHD2 gene can inhibit the expression of the GBM mesenchymal marker genes CHI3L1 and CD44 (Figure 9A–9D). Further research on MES marker proteins. In our study, we found that CD44, BMI1 and ZEB1 showed different degrees of expression inhibition after FCGR2A and EHD2 were silenced, but VIM did not change significantly (Figure 9E, 9F). We further verified that these genes play an important role in glioma.

## DISCUSSION

Human brain glioma is the most common primary malignant tumor. But the prognosis for patients with these tumors remains poor despite standard care of surgery, radiation therapy and temozolomide chemotherapy [19]. Therefore, it is imperative to identify potential prognostic and therapeutic targets for glioma. For those facing prognostic evaluation and treatment, many genes have been explored as biomarkers [20], but they are still not enough to predict the convoluted prognosis of glioma alone.

MES is a type of glioblastoma that can be transformed from other types of gliomas [21, 22]. The MES transition can be induced by master transcription factors (TFs), STAT3, C/EBPb, and TAZ through the NF-kb pathway [23]. MES is the most malignant glioblastoma and has a worse prognosis [24]. On the one hand, it has stronger resistance to treatment than other types of gliomas [25]. This therapeutic resistance may be related to the high expression of inflammation-related genes and NF-kB activation [8], which is consistent with our results. On the other hand, MES is related to recurrent tumors; in recurrent tumors, classic markers of mesenchymal tissues were upregulated, including CHI3L1, CD44, and STAT3 [22]. Moreover, MES is also related to a growth advantage conferred by either a rapid rate of cell division or enhanced survival of tumor cells afforded by neovascularization [22, 26]. In short, MES is often associated with poor prognosis. Therefore, considering the malignant behaviors of MES GBMs and the analysis of the different results in this paper, we selected MES-related genes as factors to evaluate prognosis.

As previously reported using a model, immune-related genes were successfully selected to predict the prognosis of glioma with a protein expression and mRNA data set from The Cancer Genome Atlas [27]. At the same time, m^6^A methylation, immune-related lncRNA and amino acid metabolism-related genes were used to assess the prognosis of glioma [28–30]. Therefore, we used this model to explore whether MES-related genes play a more important role in the prognostic evaluation of glioma. The identification of 21 intersecting genes related to prognosis in the TCGA and Ivy GAP databases was performed as the first step by using the mesenchymal gene set. Then, fifteen genes that were differentially expressed between normal and glioma samples were analyzed and found to be upregulated in the poor prognosis group. Based on a highly reliable survival model, consensus clustering and Lasso regression, which are widely used to generate prognostic genes in the context of high-dimensional data, were applied in this study. We first used the univariate Cox model to screen OS-related genes and applied the Lasso regression model to obtain the regression coefficients. The six genes were obtained to construct a risk score model from the Lasso regression coefficients [31]. The integration of multiple gene markers into a single model was used because the predictive performance of such models can be improved compared to that of a single predictive biomarker. The study found that the risk score model constructed based on TCGA can accurately predict the prognosis of glioma.

Hypoxia-inducible factor-1α (HIF-1α), as the main transcription factor during hypoxia, is significantly upregulated in the MES subtype and plays an important role in tumor angiogenesis and proliferation [32]. Inflammation-related genes are also highly expressed in the MES subtype [8, 33–35]. Epithelial-to-mesenchymal transition is a common pattern of increased malignant behavior and disease progression of epithelial tumors, and the MES phenotype is closely related to this change [25]. This finding supports our GSEA results. Compared with the low-risk group, the high-risk group showed enrichment in the biological processes of epithelial-mesenchymal transition, angiogenesis, inflammation and hypoxia. Similarly, GO and KEGG analyses showed that MES-related genes are closely related to cell adhesion-related processes. These processes are closely related to cancer progression and involve EMT, tumor angiogenesis and the development of an inflammatory tumor microenvironment [35–37]. Consequently, MES-related genes are associated with a higher degree of malignancy and poorer prognosis in gliomas.

Although studies have confirmed that the six selected genes used to construct the signature are related to glioma, they are functionally independent of each other. In particular, EMP3 has been found to upregulate the surface expression of αvβ3 integrin, activate focal adhesion kinase and Src kinase, promote cell migration and invasion, promote tumor growth *in vivo*, and serve as a prognostic evaluation factor for glioma [38, 39]. PLAUR can promote cell survival, migration and resistance to targeted cancer therapeutics in cultured glioblastoma cells, and PLAUR expression is inversely proportional to patient survival [40]. MVP enhances glioma aggressiveness through the epidermal growth factor receptor (EGFR)/phosphatidylinositol 3-kinase (PI3K) signaling axis [41]. Moreover, compared with normal tissues, RRAS is highly expressed in gliomas. Its overexpression is associated with the early stage of cancer and positively related to the degree of malignancy [42]. The remaining two genes, FCGR2A and EHD2, have not been studied in gliomas. In our research, we found that silencing these genes can inhibit the proliferation, migration and invasion of glioma cells. Simultaneously, silencing these genes suppresses the expression of mesenchymal marker genes. Overall, the six genes used to construct the risk coefficient model promote the proliferation, migration and invasion of glioma by affecting the EMT process, indicating that they are of particular significance for the prognostic evaluation and treatment of glioma.

In summary, we identified MES-related genes that can be distinguished from the clinical and molecular characteristics of glioma. We believe these 6 MES-related genes are potential prognostic markers or therapeutic targets for glioma patients. Furthermore, we developed a six-MES-related gene expression-based risk signature that could better predict OS for glioma. Functional analysis was carried out, and the effects of two of the genes on the proliferation, migration and invasion of glioma were studied. Nonetheless, the exact mechanism of how these MES-related genes impact the prognosis of glioma is still obscure. Therefore, further research to clarify the underlying mechanism of MES-related genes in glioma is urgently needed.

## MATERIALS AND METHODS

### Data sources

Mesenchymal-related gene sets were downloaded from TCGA (https://xena.ucsc.edu) and Ivy GAP (http://glioblastoma.alleninstitute.org). 1108 normal brain tissue samples were downloaded from GTEx, and patients with glioma were downloaded from the TCGA data portal as a training set, which contained 650 glioma samples after excluding incomplete cases. These data include matched clinical information such as age, gender, survival time and cancer type. To from the testing set, data points with insufficient clinical information were removed from the CGGA (http://www.cgga.org.cn/index.jsp) database, and 280 clinical data points in total were obtained. The workflow of this study is shown in Supplementary Figure 1.

### Consensus clustering

We carried out genetic difference analysis with the R programming language (http://cran.r-project.org) to obtain the intersecting genes of MES-related genes from the Ivy GAP and TCGA datasets. Twenty-one overlapping MES-related genes were selected, and then differentially expressed genes were identified between normal and glioma samples. Finally, a data set of 15 significant genes was obtained from 21 genes. We used the R package "Consensus Cluster Plus" to perform consensus clustering. The cumulative distribution function (CDF) and consensus matrix were used to evaluate the optimal number of subgroups.

### Bioinformatics analysis

We used univariate and multivariate Cox analyses to screen prognosis-related genes and assess the prediction model of independent prognostic factors. The R programming language was used to perform Lasso regression analysis to obtain the risk score. Through the regression coefficient weighted by the linear combination of genes, the risk score was obtained, and the high-risk group and the low-risk group were obtained based on the median risk score. Kaplan-Meier survival curves were used to analyze the relationship between different groups and survival time. ROC curves were used to judge the sensitivity and specificity of the predictive models to assess prognosis with the R package.

### Functional analysis

The hallmark gene sets, which are part of the MSigDB gene set, were downloaded from the Molecular Signatures Database. Gene set enrichment analysis (GSEA) was used to reveal the signaling pathways most likely to be affected by the six MES-related genes by using GSEA software (4.0.1) (http://www.broadinstitute.org/gsea/index.jsp). Gene Ontology (GO) and Kyoto Encyclopedia of Genes and Genomes (KEGG) analyses used the R programming language to study the cell functions associated with the risk factors composed of six genes in the TCGA database.

### Cell and cell culture

Human glioma cell lines including LN18, U251, T98G, SNB19 and SF126, which were all purchased from the Chinese Academy of Sciences (Shanghai, China). Glioma cells were cultured in Dulbecco’s Modified Eagle’s medium (DMEM; Hyclone, GE Healthcare Life Sciences) containing 10% fetal bovine serum (FBS, Gibco, Thermo Fisher Scientific, Inc.) in an incubator at 37° C and a carbon dioxide concentration of 5%. After the cells have grown to more than 90%, digest with 0.25% enzyme (C0201; Beyotime, China) to passage cell. Based on this culture state, the medium of the cells was changed every 2 days.

### Cell transfection

FCGR2A and EHD2 siRNAs were designed by RIBOBIO (Guangzhou RiboBio Co.). FCGR2A siRNA included three sequences (siFCGR2A-1 AGGCTGTGCTGAAACTTGA, siFCGR2A-2 GGTCATTGCGACTGCTGTA, and siFCGR2A-3 CTTCAACCATTGACAGTTT), and the EHD2 siRNA also included three sequences (siEHD2-1 AGACCAGCTTCATCCAGTA, siEHD2-2 GCACGACTTCACCAAGTTT, and siEHD2-3 TGCGAAGATTCAGCTGGAA). Then, we used the siRNAs to silence FCGR2A in LN18 and T98G cell lines and to silence EHD2 in U251 and SNB19 cell lines with the transfection reagent jetPRIME (Poly plus-transfection®). Continue to cultivate for 24 h, then change the medium.

### Total RNA isolation and quantitative real-time polymerase chain reaction (qRT-PCR)

Total RNA was extracted by TRIzol reagent (Invitrogen, Thermo Fisher Scientific) according to the manufacturer’s instructions. PrimeScript RT Master Mix (Perfect Real Time) (RR036A; Takara) was used for RNA reverse transcription, and quantitative real-time PCR (qRT-PCR) was performed with TB Green™ Premix Ex Taq™ II (Tli RNaseH Plus) (RR820A; Takara). The total volume of each PCR reaction mixture is 10ul, 45 cycles are performed, denaturation at 95° C for 15 seconds, annealing at 60° C for 30 seconds, and extension at 72° C for 30 seconds. The relative mRNA expression of each gene was calculated using the 2−ΔΔCt method. The following PCR primers were used:

FCGR2A forward: 5'-ACTATGGAGACCCAAATGTCTCAG-3'; reverse:5′-GCAAAACTGTCAATGGTTGAAGC-3'.

EHD2 forward: 5'-AACCCTTTCGGAAACACCTT-3'; reverse: 5'-TCGATGATGCTGATGCTCTC-3'.

CHI3L1 forward: 5'-CCCTGGACGGAGAGACAAAC-3'; reverse:5′-GCCTCAACATGTACCCCACA -3′.

CD44 forward: 5'-CGCCAAACACCCAAAGAAGA-3'; reverse: 5′-TTCCTGCTTGATGACCTCGT -3′.

GAPDH forward: 5'-AGCAAGAGCACAAGAGGAAG-3'; reverse:5′-GGTTGAGCACAGGGTACTTT -3′.

### Methyl thiazolyl tetrazolium (MTT) assay

By comparison, 2000 and 4000 cells were plated onto 96-well plates in triplicate for the MTT (3-(4,5-dimethylthiazol-2-yl)-2,5-diphenyltetrazolium bromide) assay. After incubation for 24 h, 36 h, 48 h and 72 h, the medium was replaced, and 10% of the medium volume of MTT dye (ST316; Beyotime, China) was added. Then, the cells were incubated for 4 h. After that, the medium was thoroughly removed, 150 μl of dimethyl sulfoxide (DMSO; D8370; Solarbio, China) was added, and the cells were shaken for 10 minutes. After it was fully dissolved, the absorbance at 570nm was measured with Varioskan LUX Multifunctional Microplate Reader (Thermo Scientific, MA, USA).

### Colony formation assay

The cells were seeded at a density of 200, 400, and 600 cells per well, repeated at least 3 times in a 6-well plate, and incubated at 37° C for seven days. After that, the cells were fixed with 4% paraformaldehyde (P0099; Beyotime, China) for 25 minutes. Next, the cells were stained with 0.5% crystal violet (C8470; Solaribio) for 15 minutes. Then, the crystal violet was absorbed as much as possible, and the excess crystal violet was gently washed off with PBS. Finally, we observed and took photos on the light microscope at ×100 magnification (Olympus IX71, Japan).

### Transwell migration assay and Matrigel invasion assay

Twenty-four-well Transwell chambers (29017037; Corning) were used for the Transwell migration assay and invasion assay. Briefly, the upper chamber contained 200 μl of serum-free DMEM, and 2×10^4^ transfected cells were added. Meanwhile, 600 μl of DMEM with 30% FBS was placed in the lower well and cultured at 37° C for 24 h, but SNB19 needs to be cultured for 36 h. Furthermore, an invasion assay was performed by using Matrigel (356234; BD Biosciences) inserts precoated for Matrigel, and 1×10^5^ cells were plated in each chamber and cultivated at 37° C for 48 h. After incubation, the chambers were fixed with 4% paraformaldehyde for 25 minutes and then stained with 0.5% crystal violet for 15 minutes. Cells remaining on the upper surface of the chamber were gently removed with a cotton swab. The remaining cells were counted under a light microscope at 100X magnification (Olympus IX71, Japan) in four random fields.

### Western blot analysis

The protein of glioma cells LN18 and U251 were isolated by RIPA buffer (P0013B; Beyotime) with protease inhibitors cocktail (C00001; TargetMol). Get an equal amount of protein sample from RIPA lysate for Western blot analysis. The protein was separated by SDS-PAGE, and then electro-transfer onto poly-vinylidene difluoride membranes (IPVH00010; Millipore). Then 5% skim milk was blocked for 1.5 hours, and then the primary antibodies were incubated overnight with anti-β-actin(1:10,000; #66009-1-Ig; Proteintech Group), anti-BMI1(1:2,000; #66161-1-Ig; Proteintech Group), anti-CD44(1:5,000; #60224-1-Ig; Proteintech Group), anti-Vimentin(1:5,000; #10366-1-AP; Proteintech Group), anti-ZEB1(1:1,000; 21544-1-AP; Proteintech Group), anti-bodies, respectively, at 4° C. Then incubate with the secondary antibody for 1 hour. Protein observation was done using ECL-Chemiluminescence kit (ECL-plus, Thermo Fisher Scientific, Inc.), and then detect the luminescence with a Protein Imager (Find-Do×6; Tanon). The relative gray level of western blot was measured by the ImageJ software for Microsoft Windows (National Institute of Health, Bethesda, MD, USA).

### Statistical analysis

GraphPad Prism 8 was used for graphing and statistical analysis. ImageJ software for Microsoft Windows was used for the cell number and clone formation counts. R programming language was used to perform the univariate and multivariate Cox regression analyses. Kaplan-Meier curves were used to statistically analyze the difference in survival between the two groups. Student's t-test and the chi-square test were used to assess differences in clinical characteristics between samples grouped by the risk score, and Pearson correlation was used to calculate correlations. Statistical significance was defined as a 2-tailed p value <0.05.

### Data availability statement

All data included in this study are available upon request by contact with the corresponding author.

## Supplementary Material

Supplementary Figures
