# Supplementary material for: Identification of a mesenchymal-related signature associated with clinical prognosis in glioma
Source: Aging (Albany NY). 2021 Apr 19;13(9):12431–55. doi: 10.18632/aging.202886 (PMC8148476; doi:10.18632/aging.202886)
Supplement: Supplementary Figures [file aging-13-202886-s001.pdf]

SUPPLEMENTARY FIGURES

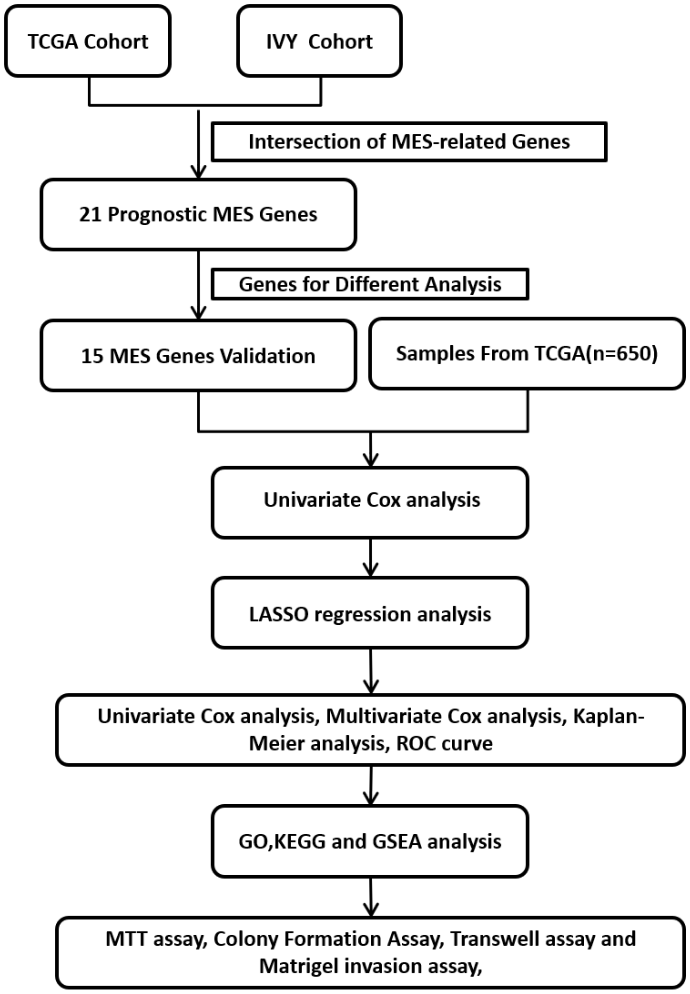

Supplementary Figure 1. The workflow of this study: data collection, analysis, and validation.

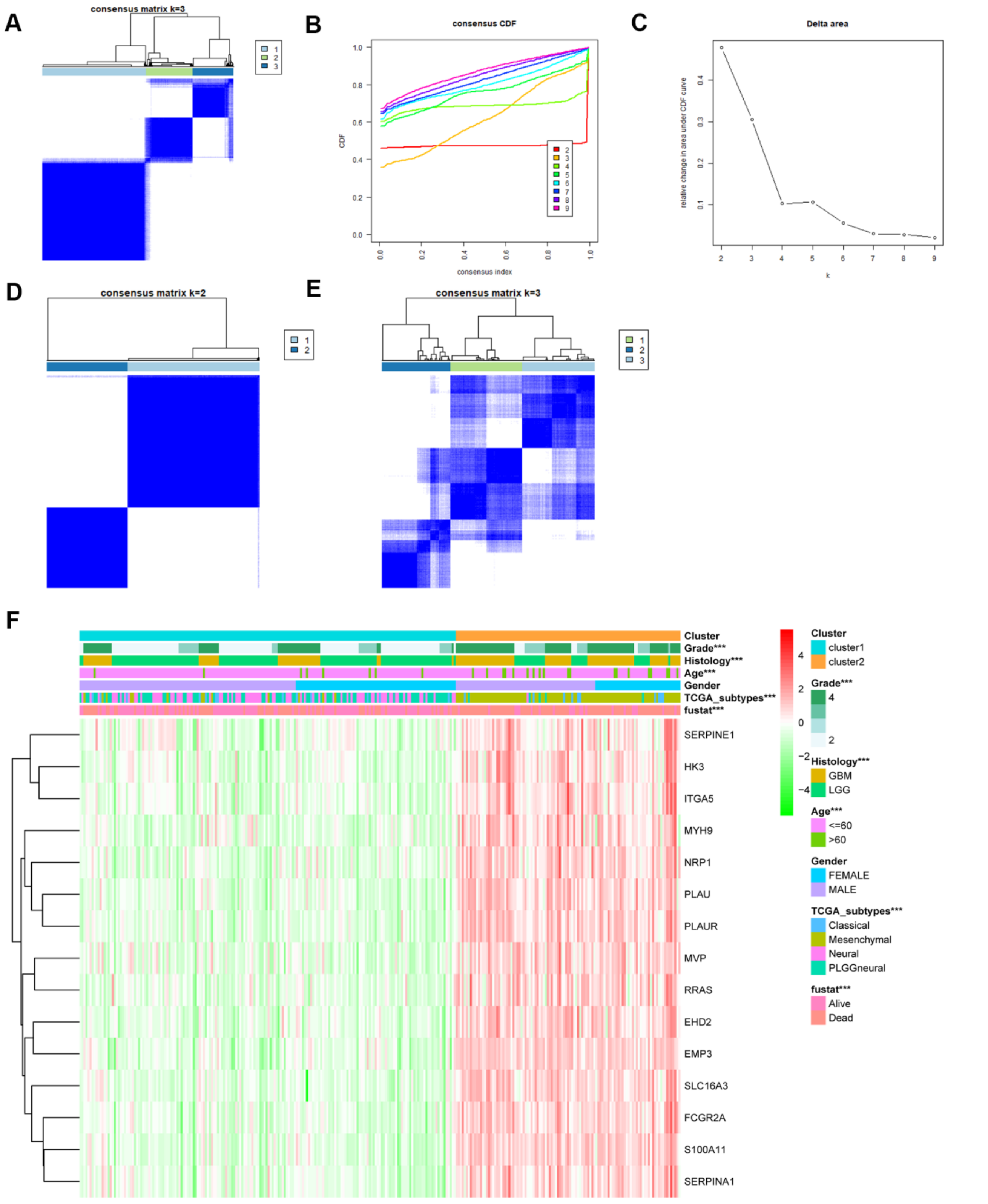

Supplementary Figure 2. Stratification and verification of glioma based on 15 MES-related genes in the CGGA cohort. (**A**) Consensus clustering matrix of 650 TCGA samples for k = 3. (**B**) Relative change in the area under the CDF curve for k = 2 to k = 9 in the testing set. (**C**) Consensus clustering CDF for k = 2 to k = 9 in the testing set. (**D**, **E**) Consensus clustering matrix of 280 CGGA samples for k = 2 and k = 3. (**F**) Heat map of MES-related genes between CGGA cluster 1 and cluster 2 used to verify consensus clustering based on TCGA. CDF, cumulative distribution function; **P <0.01; ***P <0.001.

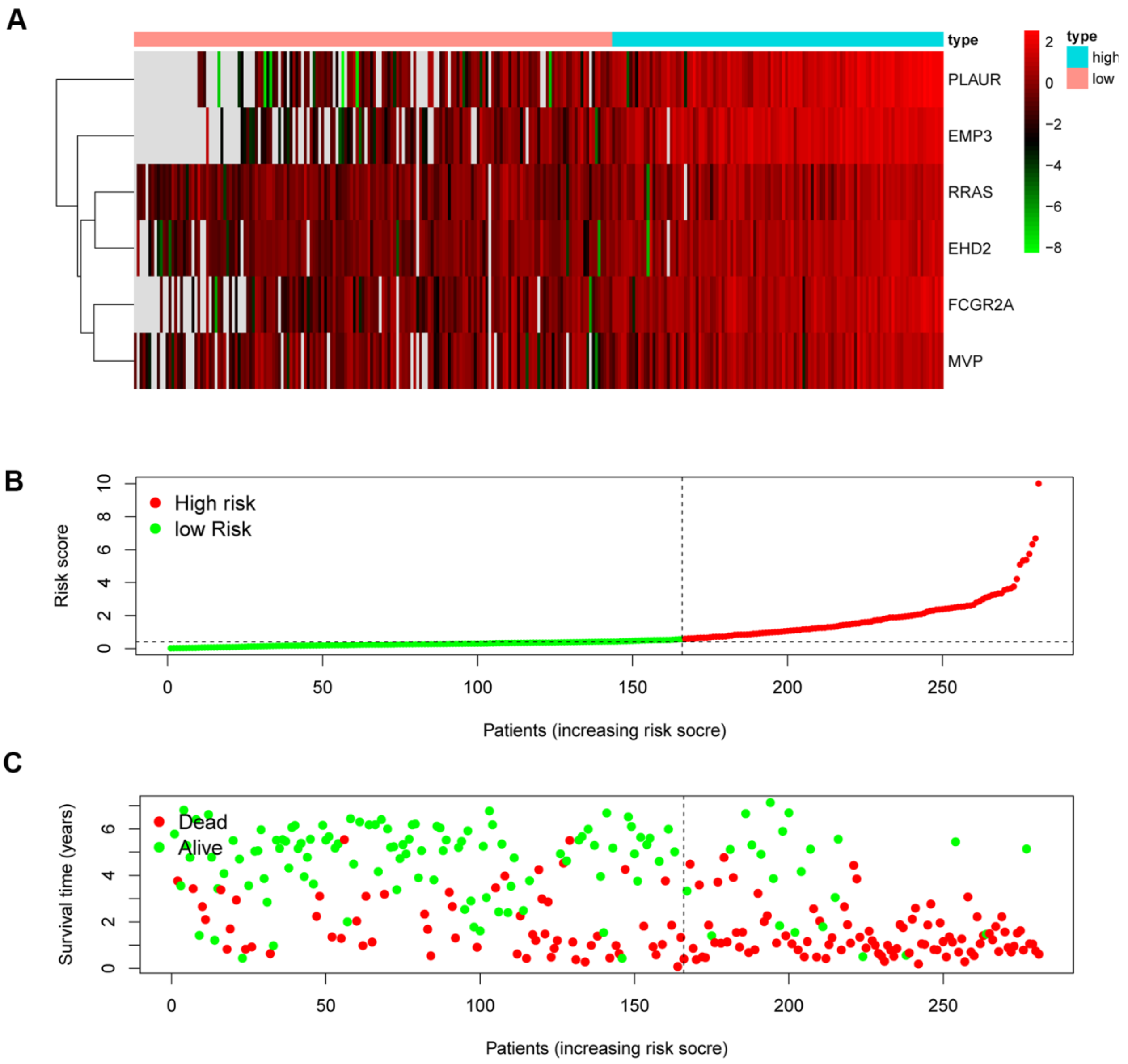

Supplementary Figure 3. Verification of the 6-gene risk signature in CGGA datasets. (**A**) Heat map shows the expression difference of six MES-related genes between the high-risk group and the low-risk group. (**B**) Risk curve represents the risk score and distribution of 280 cases from the CGGA database. (**C**) The survival status graph shows the difference in survival time of 280 cases from the CGGA database (each point represents a sample, **B**, **C**).

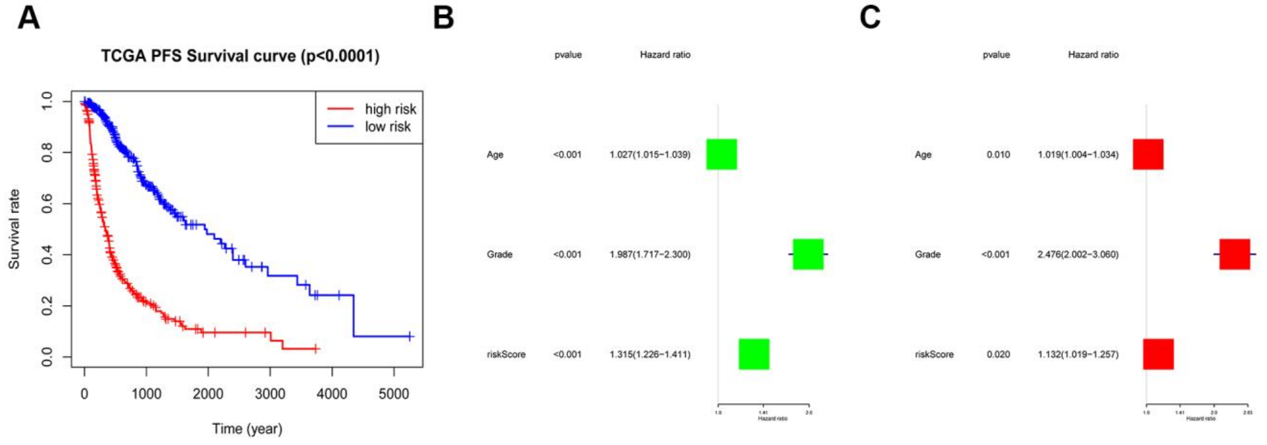

Supplementary Figure 4. CGGA confirms the independent prognostic value of the risk score. (**A**) Progression-free survival analysis between the high- and low-risk groups in the TCGA cohort. (**B**) Univariate Cox regression analysis of clinical pathologic features for OS in the CGGA cohort. (**C**) Multivariate Cox regression analysis of clinical pathologic features for OS in the CGGA cohort.

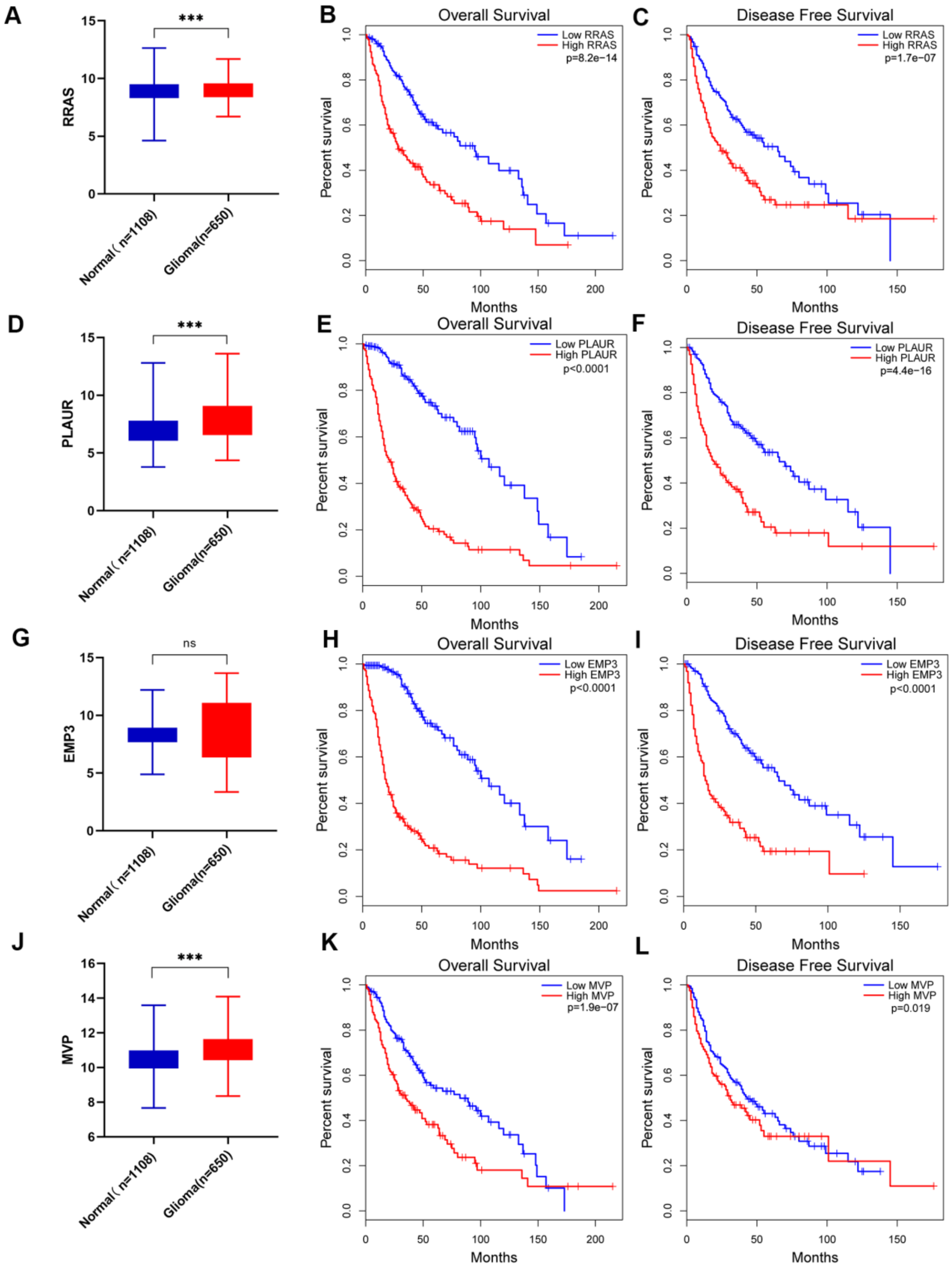

Supplementary Figure 5. RRAS, PLAUR, EMP3 and MVP gene expression differences and survival rate differences. (**A**) Differences in RRAS expression between the normal group and the glioma group from the TCGA and GTEX data. (**B**, **C**) Overall survival analysis (**B**) and disease-free survival analysis (**C**) of the relationship between RRAS expression level and survival time from the TCGA database. (**D**) Differences in PLAUR expression between the normal group and the glioma group from the TCGA and GTEX data. (**E**, **F**) Overall survival analysis (**E**) and disease-free survival analysis (**F**) of the relationship between PLAUR expression level and survival time from the TCGA database. (**G**) Differences in EMP3 expression between the normal group and the glioma group from the TCGA and GTEX data. (**H**, **I**) Overall survival analysis (**H**) and disease-free survival analysis (**I**) of the relationship between EMP3 expression level and survival time from the TCGA database. (**J**) Differences in MVP expression between the normal group and the glioma group from the TCGA and GTEX data. (**K**, **L**) Overall survival analysis (**K**) and disease-free survival analysis (**L**) of the relationship between MVP expression level and survival time from the TCGA database. Ns: no significance; ***P<0.001.

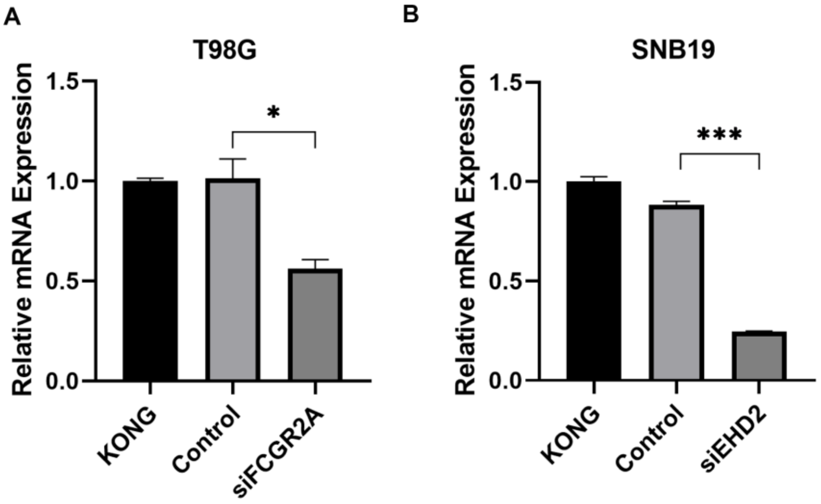

Supplementary Figure 6. The effect of FCGR2A being silenced by siFCGR2A (pro) in T98G (**A**) and the effect of EHD2 being silenced by siEHD2 (2) in SNB19 (**B**). KONG stands for untreated cell; Control stands for Negative control group. *P<0.05; ***P<0.001.

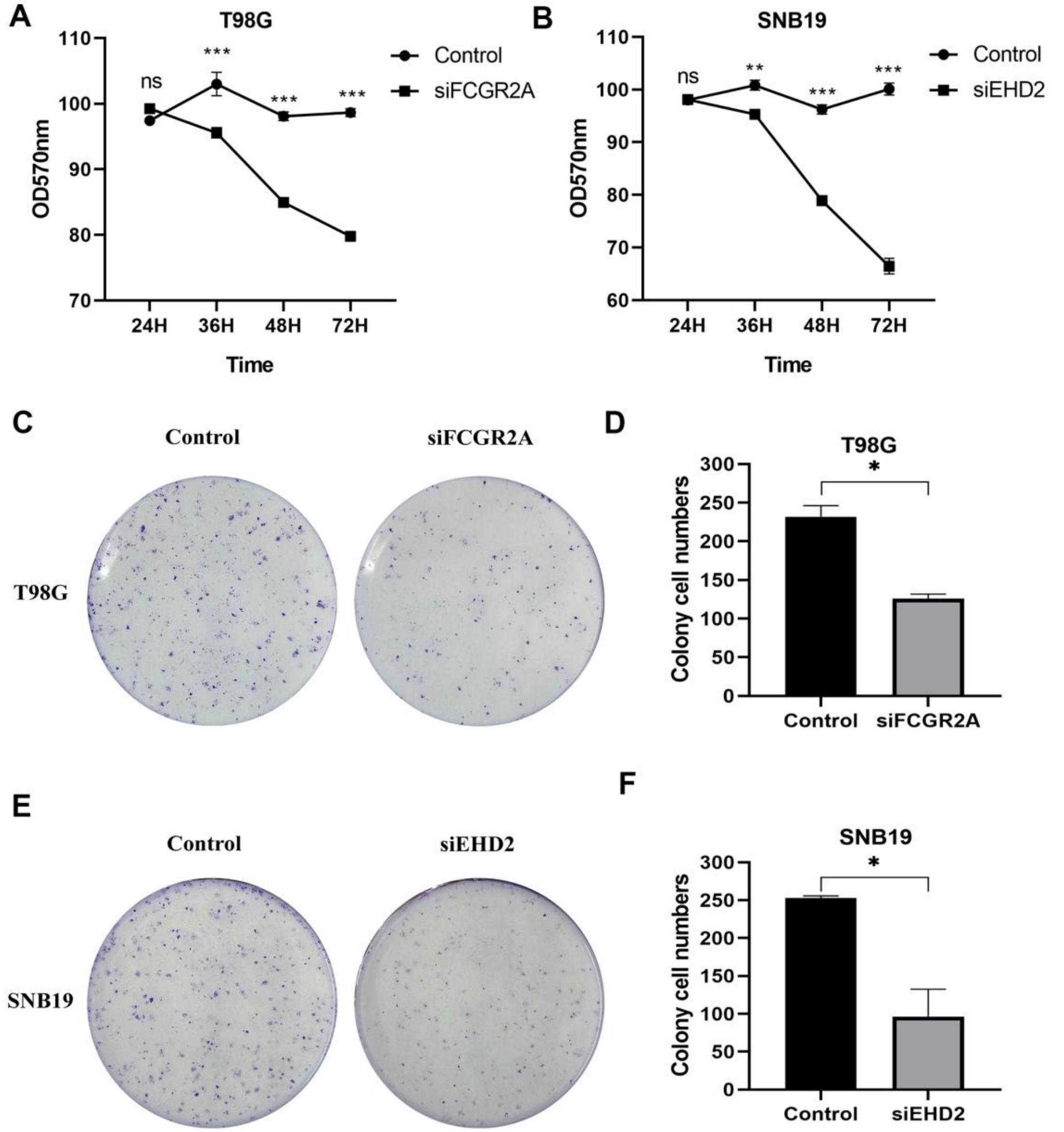

Supplementary Figure 7. The effect of glioma cell clone and proliferation ability after silencing FCGR2A or EHD2. (**A**, **B**) Cell proliferation was measured by MTT assay for 24 hours up to 72 hours. (**C**, **D**) Representative imaging (**C**) or counting (**D**) of the colonies formed by T98G cells after silencing with FCGR2A for 9 days. (**E**, **F**) Representative imaging (**E**) or counting (**F**) of the colonies formed by SNB19 cells after silencing with EHD2 for 7 days. Ns: no significance; *P<0.05; **P<0.01; ***P<0.001.
